# A protocol of systematic review and meta-analysis of acupuncture for drug resistant epilepsy

**DOI:** 10.1097/MD.0000000000021073

**Published:** 2020-07-10

**Authors:** Ze-Yu Wang, Yao-Jia Jiang, Zeng-Mian Wang, Ming-Yu Ren

**Affiliations:** Third Ward of Neurology Department, First Affiliated Hospital of Jiamusi University, Jiamusi, China.

**Keywords:** acupuncture, drug resistant epilepsy, effectiveness, safety

## Abstract

**Background:**

This study aims to appraise the effectiveness and safety of acupuncture for drug resistant epilepsy (DRE).

**Methods:**

We will search all potential randomized controlled trials (RCTs) of acupuncture for patients with DRE from their origin to March 1, 2020: MEDLINE, EMBASE, Cochrane Library, CINAHL, Scopus, WANGFANG, and Chinese Biomedical Literature Database. We will not apply any restrictions to the language and publication date. All RCTs investigating the effectiveness and safety of acupuncture for patients with DRE will be included. Study quality will be appraised by Cochrane risk of bias, and statistical analysis will be scrutinized by RevMan 5.3 software. Whenever possible, a narrative summary to describe study quality and content of the evidence will be performed.

**Results:**

This study will provide summarize high quality evidence and will utilize a variety of outcome measurements to verify effectiveness and safety of acupuncture for DRE.

**Conclusion:**

The results of this study will seek to explore the effectiveness and safety of acupuncture for DRE.

**Systematic review registration:**

PROSPERO CRD42020170517.

## 1 Introduction

Epilepsy is a chronic neurological disease that is caused by a variety of factors.^[1,2]^ It is characterized by recurrent, episodic and temporary neurological function impairments because of the excessive discharge of brain neurons.^[3]^ Epidemiological studies reported the prevalence rate of epilepsy is about 1%.^[4]^ Despite numerous antiepileptic drugs are available for the management of epilepsy, there are more than 30% patients progressing to drug-resistant epilepsy (DRE),^[5]^ which causes increasing morbidity and mortality.^[6–8]^

Acupuncture has been utilized in treating a variety of disorders, such as pain, allergic rhinitis, post-stroke spastic hemiplegia, fertilization, cardiac arrhythmia, migraine, perimenopausal depressive disorder, overactive bladder, uterine fibroids, urinary incontinence, hiccups, and epilepsy.^[9–31]^ Although published systematic reviews assessed the effectiveness and safety of acupuncture for the treatment of patients with epilepsy,^[27,30]^ no study has specifically addressed its efficacy and safety for the treatment of patients with DRE. In addition, an increasing number of clinical trials focus on acupuncture in treating DRE.^[32–46]^ Thus, this systematic review will specifically investigate the effectiveness and safety of acupuncture in treating DRE.

## 2 Methods

### 2.1 Study registration

This study was funded and registered on PROSPERO (CRD42020170517). We report it based on the guidelines of the preferred reporting items for systematic reviews and meta-analysis (PRISMA) Protocol statement.^[47–48]^

### 2.2 Dissemination and ethics

We will publish it on a peer-reviewed journal or a conference meeting. This study will not extract individual patient data, thus no ethic approval is needed.

### 2.3 Study eligibility criteria

#### 2.3.1 Types of studies

All randomized controlled trials (RCTs) of acupuncture for the treatment of patients with DRE will be included regardless language and publication date. However, we will not consider literatures of animal studies, case report, case series, uncontrolled studies, non-clinical trials, non-RCTs, and quasi-RCTs.

#### 2.3.2 Types of participants

Any patients who were diagnosed as DRE will be included irrespective country, race, age, gender, and duration and severity of DRE.

#### 2.3.3 Types of interventions

##### 2.3.3.1 Experimental interventions

All patients in the experimental group received any forms of acupuncture therapy, such as scalp acupuncture, manual acupuncture, electroacupuncture and dry acupuncture.

##### 2.3.3.2 Control interventions

As a control therapy, patients could use any treatments, such as oral medication, surgery, and mindfulness-based therapy. However, we will not consider treatments involving any forms of acupuncture treatment as a comparator.

#### 2.3.4 Type of outcome measurements

##### 2.3.4.1 Primary outcome

Seizure freedom (defined as no seizures attack after treatment within a period that corresponds to three times the longest pre-treatment inter-seizure interval during the past year).

##### 2.3.4.2 Secondary outcome

Frequency of seizures (times/per week or times/per month),
Quality of life (measured by any related and validated scales, such as 36-Item Short Form Survey),
Emergency visits (times/per week or times/per month), and
Adverse events.

### 2.4 Search strategy and data management

#### 2.4.1 Search strategy

We will search all potential RCTs on acupuncture in treating DRE in below electronic databases from origin to March 1, 2020 without language and publication date limitations: MEDLINE, EMBASE, Cochrane Library, CINAHL, Scopus, WANGFANG, and Chinese Biomedical Literature Database. A search strategy sample with details for MEDLINE is created (Table 1). We will also modify similar search strategies for other electronic databases.

**Table 1:**
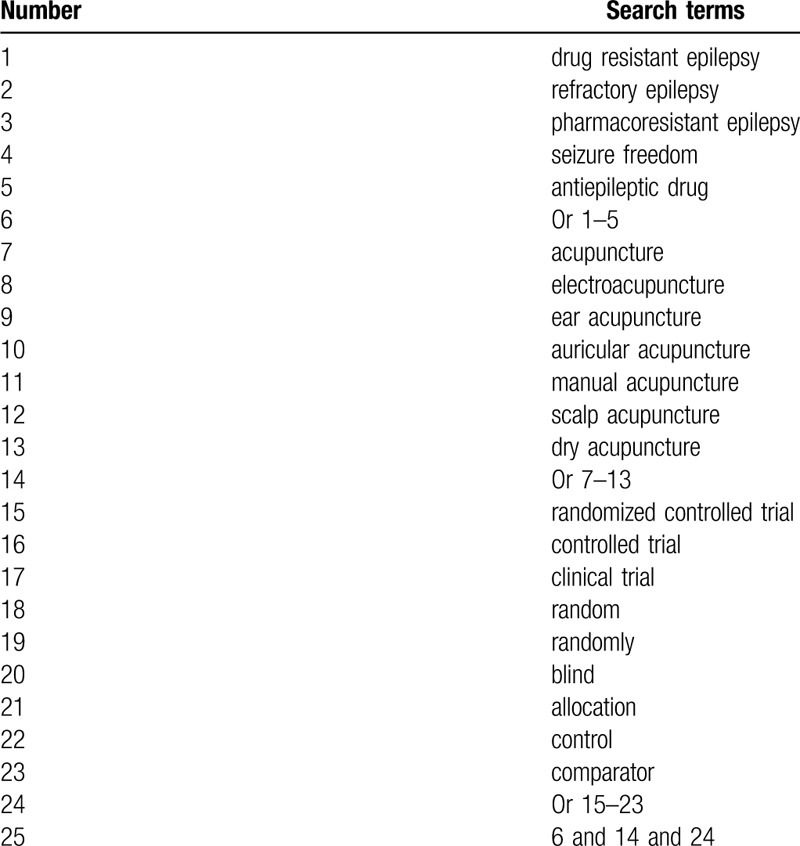
Search strategy of MEDLINE.

Meanwhile, we will examine relevant conference/meeting proceedings, and reference lists of relevant reviews to prevent missing any potential trials.

#### 2.4.2 Study selection

We will import all identified literatures into EndNote X9 software to delete any duplicates. Two authors will screen the titles/abstracts of all potential studies to remove studies that are not related to the topic. Then, full-text of remaining studies will be read carefully to further determine whether they fulfill all eligible criteria. If necessary, a third author will help to solve any divergence between 2 authors. The reasons for all excluded studies will be recorded in a table. Details of study selection will be exerted in a PRISMA flowchart.

#### 2.4.3 Data extraction and management

Two authors will independently obtain the data from all included RCTs by a standardized template sheet developed specifically for this study. Any confusion will be cleared up with the help of a third experienced author via discussion, and a consensus will be reached.

Data to be obtained from the eligible trials is as follows:

Study information: such as title, first author name, publication time, et al.
Participants: such as age, gender, severity and duration of DRE, et al.
Article methods: such as randomization specifics, blind, et al.
Interventions and comparators: such as types of treatments, dosage, frequency, et al.
Outcome indicators: such as reported outcomes and adverse events, et al.
Others: such as conflict of interest, et al.

#### 2.4.4 Dealing with missing data

We will connect primary authors to request any insufficient or missing data. If those data is not achievable, we will analyze available data only.

### 2.5 Study quality assessment

Two authors will independently appraise study quality of all included RCTs using Cochrane risk of bias tool through 7 domains. Each consideration is rated as low, unclear or high risk of bias. Any difference will be worked out by a third author through discussion.

### 2.6 Statistical analysis

We will utilize RevMan 5.3 software to synthesize and analyze the data extracted from the eligible trials. The treatment effect of dichotomous data will be expressed as risk ratio and 95% confidence intervals (CIs), and treatment effect of continuous data will be presented as mean difference or standardized mean difference and 95% CIs. Statistical heterogeneity across included trials will be examined using *I*^*2*^ test. *I*^*2*^ ≤ 50% manifests acceptable heterogeneity, and a fixed-effects model will be employed; while *I*^2^ >50% implies considerable heterogeneity, and a random-effects model will be exploited. If there is reasonable heterogeneity, we will conduct a meta-analysis when ample data is extracted from sufficient RCTs. On the other hand, a subgroup analysis will be explored to identify any possible sources of obvious heterogeneity. Under such situation, if it is impossible to perform meta-analysis, we will carry out a narrative synthesis to explain the findings.

#### 2.6.1 Subgroup analysis

Subgroup analysis will be carried out to find out the source of obvious heterogeneity according to different study characteristics, trial quality, interventions and controls, and outcome indicators.

#### 2.6.2 Sensitivity analysis

Sensitivity analysis will be carried to test the stability of study findings by excluding low quality trials.

#### 2.6.3 Reporting bias

If adequate numbers of RCTs are included, a funnel plot and Egger's regression test will be performed to examine any reporting biases.^[49,50]^

#### 2.6.4 Quality of evidence

The quality of evidence of each outcome indicator will be appraised by two independent authors using Grading of Recommendations Assessment Development and Evaluation.^[51]^ Any disagreements will be solved by a third author through discussion.

## 3 Discussion

DRE is a very tricky health problem that perplexes people worldwide.^[1–3]^ Although the treatment schedules of DRE are progressed, its incidence is still growing. Acupuncture has been utilized on the treatment of DRE in China for many years.^[30–46]^ Despite previous systematic reviews conducted the effectiveness and safety of acupuncture in treating epilepsy,^[27,30]^ we do not identify systematic review that specifically focus on acupuncture in treating DRE. Thus, it is very important to perform present study.

In this study, we attempt to carry out a systematic review to provide high-quality evidence for the effectiveness and safety of acupuncture in treating DRE. We hope this study will supply more options for patients, clinician, and future studies. However, this study may still have several limitations. First, there may be insufficient number of eligible trials. Second, the sample size of included trials may be small. Third, most of included studies may have poor methodological quality. All those limitations may affect the findings of this study.

## Author contributions

**Conceptualization:** Ze-Yu Wang, Yao-Jia Jiang, Ming-Yu Ren.

**Data curation:** Zeng-Mian Wang.

**Formal analysis:** Ze-Yu Wang, Yao-Jia Jiang, Zeng-Mian Wang, Ming-Yu Ren.

**Investigation:** Ze-Yu Wang.

**Methodology:** Yao-Jia Jiang, Zeng-Mian Wang, Ming-Yu Ren.

**Project administration:** Ze-Yu Wang.

**Resources:** Yao-Jia Jiang, Zeng-Mian Wang, Ming-Yu Ren.

**Software:** Yao-Jia Jiang, Zeng-Mian Wang, Ming-Yu Ren.

**Supervision:** Ze-Yu Wang.

**Validation:** Ze-Yu Wang, Yao-Jia Jiang, Zeng-Mian Wang, Ming-Yu Ren.

**Visualization:** Ze-Yu Wang, Yao-Jia Jiang, Zeng-Mian Wang, Ming-Yu Ren.

**Writing – original draft:** Ze-Yu Wang, Yao-Jia Jiang, Zeng-Mian Wang, Ming-Yu Ren.

**Writing – review & editing:** Ze-Yu Wang, Yao-Jia Jiang, Ming-Yu Ren.
